# Metabolomic Analysis Reveals Changes in Plasma Metabolites in Response to Acute Cold Stress and Their Relationships to Metabolic Health in Cold-Acclimatized Humans

**DOI:** 10.3390/metabo11090619

**Published:** 2021-09-12

**Authors:** Zuzana Kovaničová, Miloslav Karhánek, Tímea Kurdiová, Miroslav Baláž, Christian Wolfrum, Barbara Ukropcová, Jozef Ukropec

**Affiliations:** 1 Institute of Experimental Endocrinology, Biomedical Research Center, Slovak Academy of Sciences, 84505 Bratislava, Slovakia; zuzana.kovanicova@savba.sk (Z.K.); miloslav.karhanek@savba.sk (M.K.); timea.kurdiova@savba.sk (T.K.); miroslav.balaz@hest.ethz.ch (M.B.); barbara.ukropcova@savba.sk (B.U.); 2 Institute of Food, Nutrition and Health, ETH Zurich, 8603 Schwerzenbach, Switzerland; christian-wolfrum@ethz.ch; 3 Institute of Pathophysiology, Faculty of Medicine, Comenius University, 81108 Bratislava, Slovakia

**Keywords:** metabolomics, plasma metabolome, cold exposure, non-shivering thermogenesis, cold acclimatization, brown adipose tissue

## Abstract

Cold exposure results in activation of metabolic processes required for fueling thermogenesis, potentially promoting improved metabolic health. However, the metabolic complexity underlying this process is not completely understood. We aimed to analyze changes in plasma metabolites related to acute cold exposure and their relationship to cold-acclimatization level and metabolic health in cold-acclimatized humans. Blood samples were obtained before and acutely after 10–15 min of ice-water swimming (<5 °C) from 14 ice-water swimmers. Using mass spectrometry, 973 plasma metabolites were measured. Ice-water swimming induced acute changes in 70 metabolites. Pathways related to amino acid metabolism were the most cold-affected and cold-induced changes in several amino acids correlated with cold-acclimatization level and/or metabolic health markers, including atherogenic lipid profile or insulin resistance. Metabolites correlating with cold-acclimatization level were enriched in the linoleic/α-linolenic acid metabolic pathway. N-lactoyl-tryptophan correlated with both cold-acclimatization level and cold-induced changes in thyroid and parathyroid hormones. Acute cold stress in cold-acclimatized humans induces changes in plasma metabolome that involve amino acids metabolism, while the linoleic and α-linolenic acid metabolism pathway seems to be affected by regular cold exposure. Metabolites related to metabolic health, thermogenic hormonal regulators and acclimatization level might represent prospective molecular factors important in metabolic adaptations to regular cold exposure.

## 1. Introduction

Cold exposure results in stimulation of defense mechanisms aimed at maintaining body temperature by reducing heat loss and promoting heat production by muscle shivering or non-shivering thermogenic mechanisms in brown fat, white fat and skeletal muscle. Currently, a great deal of attention is aimed at understanding the role of cold-induced facultative and adaptive thermogenesis in human energy metabolism, and to what extent it is possible to modulate the process in the long run by cold acclimatization, exercise or pharmacology [1]. During cold exposure, the high metabolic/thermogenic activity of brown fat can be demonstrated by the accelerated uptake and utilization of various metabolic substrates including glucose, non-esterified fatty acids, succinate and acetate or lactate in parallel with increased facultative energy expenditure [2,3]. The non-shivering production of heat typically requires the onset of lipolysis for the efficient substrate supply of free fatty acids or their derivates from white or brown adipose tissues [4,5]. The necessity of inter-organ crosstalk in the efficient substrate supply of the thermogenic process was shown by the flux of metabolites between white and brown adipose tissue and the liver [6]. Therefore, it is important to further assess the complexity of the metabolic processes activated during cold exposure. Understanding of these regulations could provide knowledge necessary for targeted treatment of metabolic diseases such as obesity, dyslipidemia and type 2 diabetes using the therapeutic potential of brown fat.

Recent studies have shown the effects of acute cold exposure on changes in brown adipose tissue metabolome in comparison to other fat depots in mice [3,7,8] and on changes in circulating polyunsaturated fatty acids and oxylipins in humans [9]. However, the effects of acute cold stress on the broader spectrum of metabolites in humans are not well described. In this study, we aimed to explore the regulation of metabolites that could be associated with the thermogenic process by measuring plasma metabolome from cold-acclimatized individuals in response to an acute bout of cold stress induced by ice-water swimming using untargeted metabolomics. This approach allowed us not only to measure relative changes in the pool of hundreds of circulating metabolites, but also to identify pathways regulated in response to cold stress that might reveal robust changes in the whole-body energy metabolism that promote efficient production of heat. Furthermore, we aimed to investigate metabolites potentially linking the acute response to cold with the individual metabolic phenotype by evaluating associations between the regulation of metabolite levels and selected markers of metabolic health as well as thyroid and parathyroid hormones that are potentially involved in the regulation of cold-induced thermogenic process in humans [10].

## 2. Results

### 2.1. Changes in Plasma Metabolome in Response to Acute Cold Stress 

**Figure 1:**
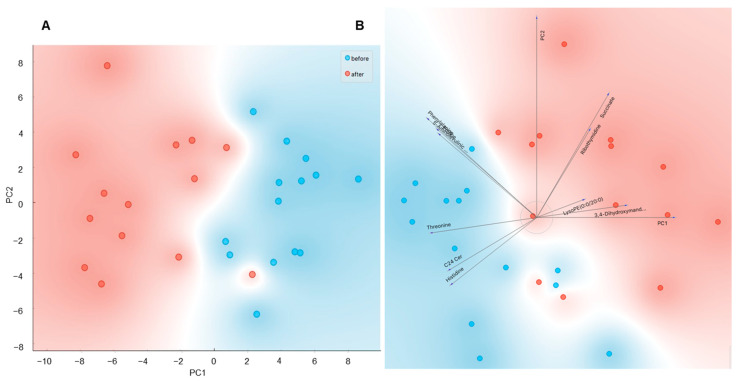
The principal component analysis (PCA) of plasma metabolome before and after ice-water swimming, explaining 50.1% of variance, PC2 = 15.4%, showing (**A**) score plot of the PCA from all 70 significantly regulated metabolites and (**B**) loading plot (biplot) of the first 10 most significantly changed metabolites. The effect of ice-water swimming was analyzed using Wilcoxon matched pairs signed-rank test with Benjamini–Hochberg correction for multiple comparison. Blue —before, red—after ice-water swimming.

**Figure 2:**
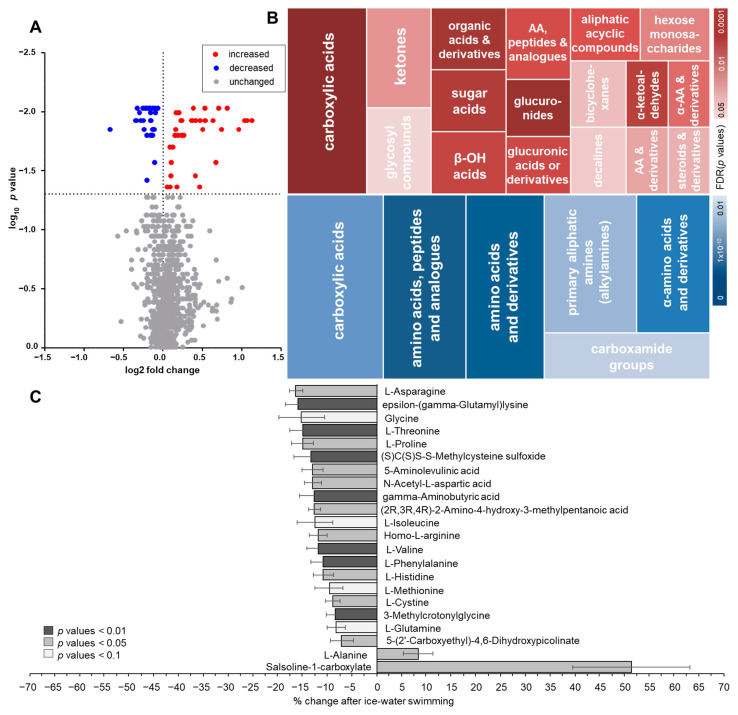
Changes in plasma metabolome in response to an acute bout of ice-water swimming characterized by (**A**) a volcano plot of the changed metabolites analyzed by Wilcoxon matched pairs signed-rank test with Benjamini–Hochberg correction and (**B**) analysis of the metabolic groups’ taxonomy enrichment of the increased (upper panel) and decreased metabolites (lower panel) based on HMDB taxonomy, performed by enrichment analysis (MBROLE 2.0) with minimum of 3 metabolites per group. Size of the tiles represents the relative percentage of matched metabolites in group, the shade of the tiles represents the significance of the enrichment (FDR-adjusted *p* values) and (**C**) analysis of ice-water swimming-induced changes in amino acids (AA) and their derivates.

Using untargeted metabolomics analysis, we were able to measure 973 metabolites in plasma from cold-acclimatized volunteers before and after an acute bout of ice-water swimming (full metabolite data list available at https://data.mendeley.com/datasets/8fyjd9yrpf/draft?a=8f865066-31e0-4dc1-a1d8-db27a7cb94e2, accessed on 10 August 2021), doi:10.17632/8fyjd9yrpf.1 [11]). Levels of 70 metabolites were significantly affected by the acute cold exposure (Table S1). A PCA analysis of the significantly regulated metabolites revealed clear distinctions between the samples taken before and after ice-water swimming (Figure 1A,B), indicating that ice-water swimming is a potent stimulus inducing complex changes in plasma metabolome. From the 70 regulated metabolites, 36 were upregulated and 34 downregulated due to the cold stress (Figure 2A). By performing enrichment analysis, we were able to identify main metabolic taxonomic classes of the regulated metabolites (Figure 2B). This revealed large heterogeneity within increased metabolites and it also pointed at major representation of amino acids and their derivates among the cold-downregulated metabolites (Figure 2B,C). In fact, we found that 11 out of 16 detected amino acids, especially the essential amino acids, were decreased or had a trend to decrease after ice-water swimming, and the only increased amino acid was alanine (Figure 2C). 

### 2.2. Pathway Analysis of Metabolome in Response to Acute Cold Stress 

**Figure 3:**
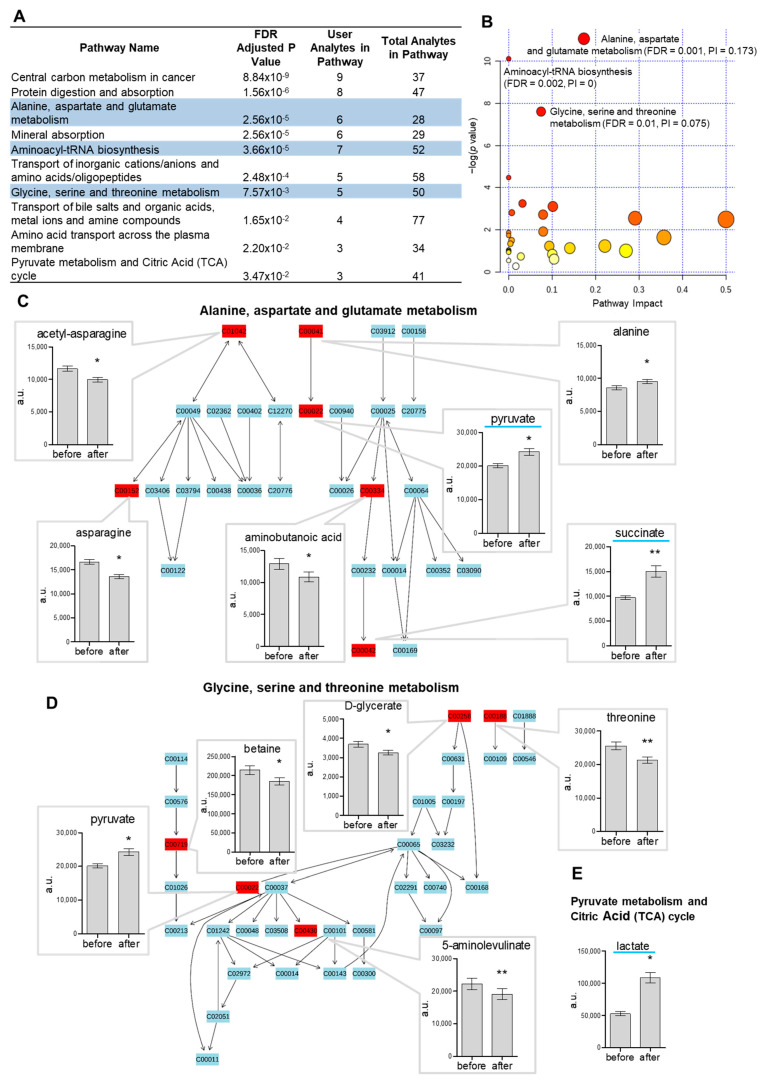
The pathway analysis of metabolome using (**A**) RaMP showing overlapping results (blue-highlighted pathways) with the analysis from (**B**) Metaboanalyst, where the 3 significantly represented pathways (FDR < 0.05) are identified, and (**C**–**E**) the top pathways are shown in detail, with the significantly regulated metabolites in plasma before/after ice-water swimming shown in bar plots (mean ± SD), * *p* < 0.05/** *p* < 0.01 analyzed by Wilcoxon matched pairs signed-rank test with Benjamini-Hochberg correction. (**C**) Alanine, aspartate and glutamate metabolism pathway, (**D**) glycine, serine and threonine metabolism pathway and (**E**) pyruvate metabolism and citric acid (TCA) cycle, where the remaining regulated metabolites pyruvate and succinate are shown in (**C**), highlighted in blue. PI—pathway impact score.

The distance map visualizes the distances between clusters of metabolites changed after ice-water swimming (Figure S1). To identify pathways modulated by cold stress, we performed pathway analysis on significantly changed metabolites (FDR-adjusted *p* < 0.05). RaMP analysis revealed 10 pathways with significant representation of regulated metabolites (Figure 3A). Pathway analysis performed in Metaboanalyst [12] confirmed significant representation of 3 out of those 10 pathways, all of which involved amino acid metabolism: (i) alanine, aspartate and glutamate metabolism (Figure 3C); (ii) aminoacyl-tRNA synthesis; and (iii) glycine, serine and threonine metabolism (Figure 3D). Metabolites involved in pyruvate metabolism and TCA cycle were also significantly affected by cold exposure (Figure 3E).

### 2.3. Role of Acclimatization Level in Cold-Induced Metabolome Changes

**Figure 4:**
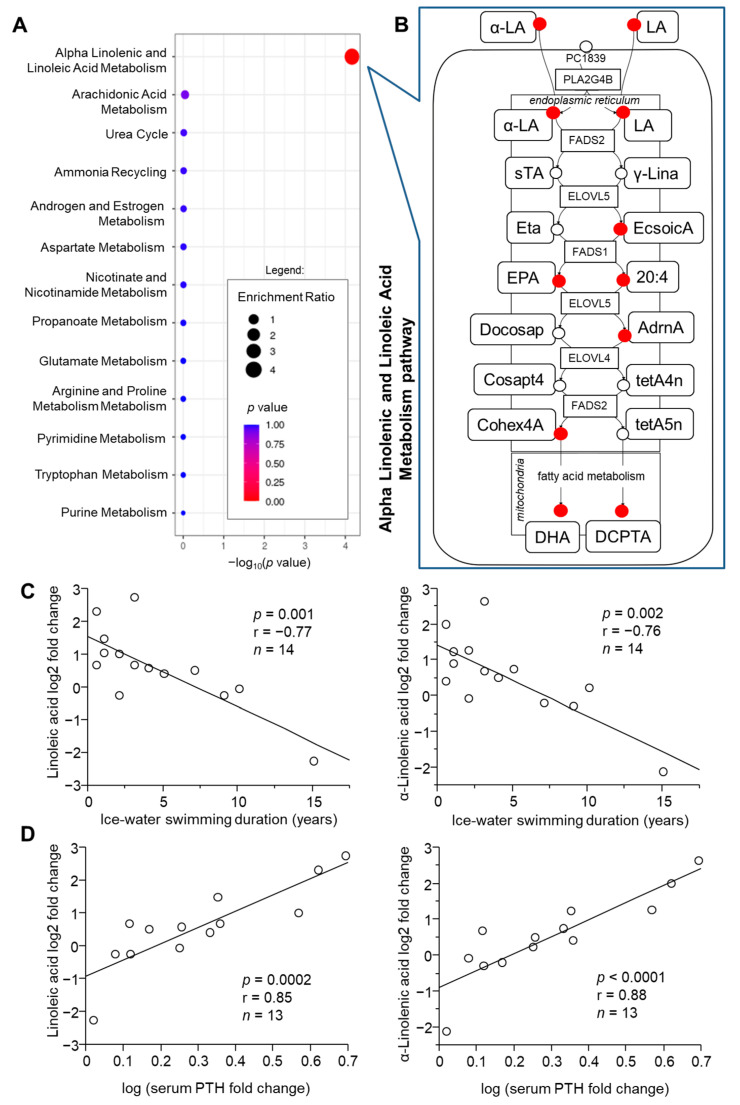
Cold-acclimatization level (ice-water swimming duration) is associated with individual changes in plasma metabolites, which are enriched in the α-linoleic (α-LA) and linolenic acid (LA) metabolism pathway. (**A**) Over-representation analysis using metabolites correlating with the duration of ice-water swimming (Pearson’s linear regression *p* < 0.05) and (**B**) the detailed representation of the pathway taken from SMPDB 2.0 database (red circles—matched correlating metabolites with Spearman’s Rho < −0.5). Cold-induced changes in α-LA and LA correlate with (**C**) the duration of the ice-water swimming habit and (**D**) with the cold-induced changes in circulating parathyroid hormone (PTH), analyzed by Pearson’s linear regression. sTA, Stearidonic acid; γ-Lina, γ-Linolenic acid; Eta, Cis-8,11,14,17-Eicosatetraenoic acid; EcsoicA, 8,11,14-Eicosatrienoic acid; EPA, eicosapentaenoic acid; 20:4, arachidonic acid; Docosap, docosapentaenoic acid (22n-3); AdrnA, adrenic acid; Cosapt4, Tetracosapentaenoic acid (24:5n-3); tetA4n, Tetracosatetraenoic acid (24:4n-6); Cohex4A, tetracosahexaenoic acid; tetA5n, tetracosapentaenoic acid (24:5n-6); DHA, docosahexaenoic acid; DCPTA, docosapentaenoic acid (22n-6).

Next, we explored whether the response of circulating metabolome to acute cold exposure could be modulated by the individual’s cold-acclimatization level indicated by the number of years dedicated to the ice-water swimming activity/sport or its weekly frequency during cold season. Using multivariate correlation analysis, we identified plasma metabolites whose cold-induced fold changes correlated with ice-water swimming duration (168) and/or frequency (92) and from these, fold changes in 13 metabolites correlated with both frequency (sessions per week within the last season) and duration (years) of ice-water swimming habit (Table S2). To evaluate whether there is any functional pattern among these metabolites, we performed enrichment analysis. We found that within the metabolites whose acute fold changes were related to the ice-water swimming habit duration, those related to linoleic and α-linolenic acid metabolism pathway were significantly enriched (Figure 4A). Pearson’s linear regression analysis confirmed that in fact, the acute cold-induced change in 9 out of 16 metabolites in the pathway correlated, all negatively, with the ice-water swimming habit duration, including α-linolenic (ALA) and linoleic acid (LA) (Figure 4A,B, Table S3). Interestingly, fold changes for eight of these fatty acid metabolites have also correlated positively with the cold stress-induced acute change in systemic level of parathyroid hormone (PTH) (Table S3). Both ALA and LA showed strong relationships with acclimatization level (Figure 4C) and cold-induced PTH change (Figure 4D). In addition, the cold-induced change in ALA correlated positively with the change in circulating thyroid stimulating hormone (TSH) levels (*n* = 13, *p* = 0.03, r = 0.59). To note, the remaining seven metabolites in the pathway were not measured by the metabolomic analysis.

### 2.4. Role of Metabolic Health in Cold-Induced Metabolome Changes

Further, we explored whether the plasma metabolome response is in any relationship to the individual anthropo-metabolic phenotypes, and found that the acute cold-induced fold changes in 38 plasma metabolites correlated with Body mass index (BMI) (Table S4), 49 metabolites correlated with the insulin resistance predictor Homeostatic Model Assessment for Insulin Resistance (HOMA-IR) (Table S5), and 155 metabolites with pro-atherogenic plasma lipid profile marker—atherogenic index (Table S6). Interestingly, the majority of the metabolites that correlated with the atherogenic index were lipids (92 out of 123 metabolites identified by MBROLE). These included several significantly enriched groups of lipids, such as prenol lipids (*n* = 29; consisting of triterpenoids (*n* = 15), monoterpenoids (*n* = 2), vitamin D and derivatives (*n* = 2) and other subclasses (*n* = 1)), lysophosphatidylcholines (*n* = 6), lysophosphatidylethanolamines (*n* = 3) and monoacyl-sn-glycerols (*n* = 3).

### 2.5. Identification of Metabolites Associated with Acclimatization Level and Metabolic Health

**Figure 5:**
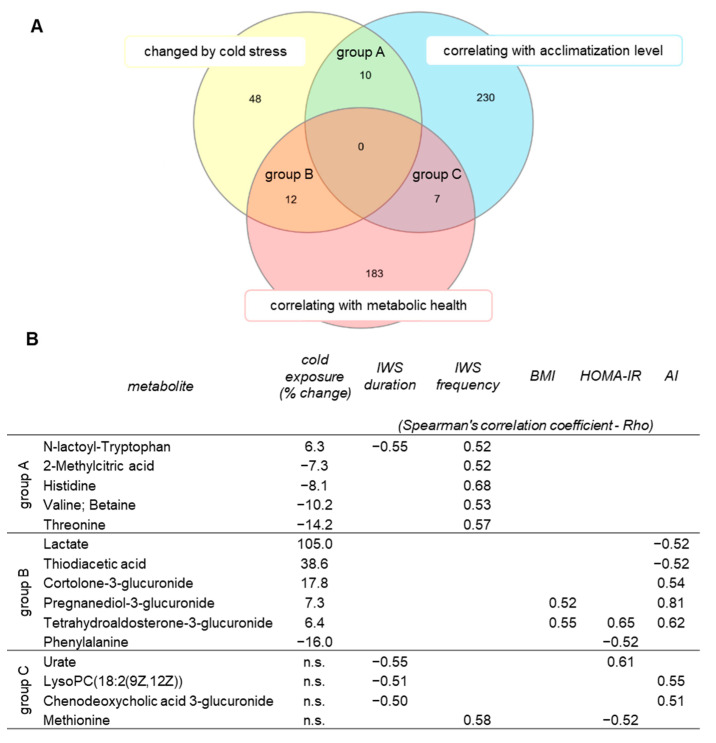
Identification of plasma metabolites changed by acute cold exposure and related to acclimatization level (IWS/ice-water swimming habit duration and/or frequency) and/or to metabolic health (BMI/body mass index, HOMA-IR/Homeostatic Model Assessment for Insulin Resistance, AI/atherogenic index), (**A**) presented in Venn diagram and (**B**) filtered to only those metabolites that are detected in humans according to The Human Metabolome Database. Acute cold-induced changes in levels of plasma metabolites were used in the associations, analyzed using Spearman’s correlation, shown are those with Spearman’s correlation coefficient (Rho) < −0.5 or > 0.5. All metabolites changed by cold exposure had *p* < 0.05 after Benjamini–Hochberg correction. n.s., not significant.

Our next analysis was aimed to identify those cold-regulated metabolites, which could be affected by the cold-acclimatization level and/or metabolic health. A set of significantly changed metabolites was compared against the metabolites whose fold changes correlated with indicators of cold-acclimatization level (ice-water swimming duration or frequency) and/or with selected markers of metabolic health (BMI, HOMA-IR, atherogenic index). Overlapping candidate metabolites are shown in the Venn diagram (Figure 5A) and the associations in Figure 5B. None of the cold-changed metabolites correlated with both acclimatization level and BMI, HOMA-IR or atherogenic index (Figure 5B). However, further correlation analysis of the cold-induced fold changes of the 70 cold-changed metabolites with an extended list of anthropo-metabolic markers revealed that 6 cold-regulated metabolites correlated with fasting insulinemia and 3 with cold-induced change in insulinemia, 12 correlated with fasting glycemia and 17 with cold-induced fold change in glycemia, 10 metabolites correlated with fasting levels of liver enzymes alanine transaminase (ALT) or aspartate transaminase (AST), 4 with high-sensitivity C-reactive protein (hsCRP) and 2 with subcutaneous adiposity and 2 with visceral adiposity (Table S7). Furthermore, we focused on those several cold-regulated metabolites that also correlated with the acclimatization level, and we found that cold-induced change in levels of N-lactoyl-Tryptophan was associated with the cold-induced changes in free thyroxine (*p* = 0.01, r = −0.68, *n* = 13) and parathyroid hormone (*p* = 0.02, r = 0.65, *n* = 13). With this approach, we have found several potential metabolites that could be important in maintaining metabolic homeostasis during cold exposure and/or those which reflect successful cold acclimatization and provide metabolic health advantage to ice-water swimmers.

## 3. Discussion

In this study, we show that ice-water swimming is a cold-stress stimulus capable of inducing systemic changes in human plasma metabolome, reflecting both the whole-body acute response to cold and chronic metabolic adaptations in cold-acclimatized individuals potentially providing them with metabolic benefits. We do not have a direct measure of the ice-water swimming-induced activation of brown fat or other thermogenic mechanisms, but we shall presume that maximal induction of thermogenesis must occur in the cold-acclimatized individuals in order to survive such intense cold stress. 

The data showed that systemic metabolic regulation is proportional to increased energy requirements and detected changes are distributed bidirectionally. The taxonomic characterization revealed that while the upregulated metabolites represent a rather heterogeneous group, a strong overrepresentation of amino acids (AA) and their derivates was found within the pool of downregulated metabolites. Lowered systemic AA content could be explained by decreased proteolysis or increased amino acid tissue uptake and utilization. However, the fact that levels of tyrosine, a marker of muscle proteolysis, were not regulated, suggests increased AA utilization. A previous report in rats indicated that cold exposure (4 °C) increased proteolysis (tyrosine release) after 6, 12 and 24 h in the soleus muscle and after 12 and 24 h it has also become apparent in the more glycolytic extensor digitorum longus muscle. Simultaneous decline in protein synthesis (tyrosine incorporation) rate was found in m. soleus and m. extensor digitorum longus in response to 24 h cold exposure [13]. However, results of another study suggest that cold does not stimulate protein degradation (urinary excretion of 3-methylhistidine) [14]. 

To discover the intricate network of metabolites regulated by cold exposure, we performed pathway analysis using the relatively new RaMP tool [15], which combines metabolomic pathways from Kyoto Encyclopedia of Genes and Genomes (KEGG), Reactome, WikiPathways and the Human Metabolome DataBase (HMDB), enabling more complex pathway analysis of the given list of metabolites in one step. Using this platform, we were able to identify 10 pathways significantly affected by cold exposure. In comparison, using the widely used Metaboanalyst tool [12], we identified three significantly affected pathways, all of which were also detected by RaMP. The most significant out of these was the alanine, aspartate and glutamate metabolism pathway with 6 out of 28 metabolites of the pathway being regulated by acute cold. Clearly, the pathways affected by acute cold mostly involved amino acid metabolism and the citric acid cycle. Interestingly, several of these metabolic pathways were also affected in brown adipose tissue (BAT) after 2–6 h of cold exposure in mice: alanine and aspartate metabolism [16]; alanine, aspartate and glutamate metabolism; glycine, serine and threonine metabolism; and pyruvate metabolism and the citric acid cycle (TCA) [8]. It is important to note that several amino acids in rodent BAT seemed to be regulated in the opposite direction when compared with the changes in human plasma related to cold exposure. For example, methionine, isoleucine and valine were all increased in BAT after 4–6 h of cold exposure [8] and serine and threonine increased in BAT after 4–6 [8] and 12 h [17] of acute cold exposure in non-acclimatized mice, but they all decreased or had a trend to decrease (isoleucine) in plasma of cold-acclimatized humans after ice-water swimming. Similarly, levels of 15 out of 19 amino acids measured after 4 h cold exposure (4 °C) were increased in BAT of wild-type mice [16]. This could indicate that the decrease in plasma amino acids may reflect their uptake and utilization by BAT in humans. However, further experiments are needed to confirm this. 

Interestingly, alanine was the only amino acid that was increased in circulation after ice-water swimming in cold-acclimatized humans. In BAT, alanine levels decreased after 6 h [8] and after 12 h of acute cold exposure [17]. Alanine levels in BAT as well as its tissue/blood concentration ratio further decreased while ALT activity increased in BAT after 15 days of cold acclimation [17], perhaps as a metabolic compensation aimed at increasing capacity for pyruvate synthesis. Interestingly, ALT gene expression was increased in human brown fat compared to white fat samples obtained at thermoneutrality [18]. Monitoring of arterial and venous blood in rat hind leg showed that alanine is released into circulation within the first 30 min of cold exposure (4 °C), which is followed by rapid uptake of alanine as well as other amino acids into tissues during the next hour [19]. In addition to glucose and fatty acids, amino acids are an important fuel source for BAT activity during cold exposure, including alanine, that is utilized in BAT from 4 h cold exposed rats [20]. Besides insulin, one of the potential factors regulating amino acid balance during cold stress is the release of catecholamines. Interestingly, tyrosine and leucine release from the gastrocnemius muscle initially dropped within the first 30 min of adrenaline perfusion and then gradually increased, peaking at 60 min after the termination of adrenaline perfusion. Alanine release, similarly to lactate, gradually increased during the 90 min adrenaline perfusion, returning to baseline within the following 90 min washout period [21].

It has been recently shown that branched-chain amino acids (BCAA) such as valine, leucine and isoleucine are decreased in response to cold exposure specifically in serum of individuals with high brown fat activity, and the magnitude of their cold-induced decrease correlated with the brown fat volume. Tracing by leucine-analogue ^18^F-fluciclovine in cold-acclimatized mice suggested increased BCAA uptake to BAT and high rate of oxidation of BCAA in BAT relative to other tissues during cold exposure. This study has further demonstrated that intact process of BCAA import and oxidation in BAT mitochondria seems to be essential for maintenance of thermogenesis as well as for prevention of diet-induced obesity and glucose intolerance [22]. We might speculate that in the case of more intense cold stimulation such as ice-water swimming, other classes of amino acids could be similarly involved in fueling of the thermogenic process in addition to the BCAA in the cold-acclimatized individuals, either directly or indirectly as precursors in the synthesis of pyruvate, which was significantly increased, or creatine (not measured), an important alternative source of thermogenesis. 

The large number of metabolites whose magnitude of response to cold was significantly associated with the ice-water swimming duration (acclimatization level) indicates an adaptive component in the metabolic processes potentially linked to the thermogenic process. Quite interestingly, this included LA, ALA and their metabolites produced in the linoleic and alpha-linolenic acid pathway. Specifically, cold-induced change in LA, ALA, 8,11,14-Eicosatrienoic acid (also known as dihomo-gamma-linolenic acid (DGLA)), eicosapentaenoic acid (EPA), arachidonic acid, adrenic acid, tetracosahexaenoic acid, docosahexaenoic acid and docosapentaenoic acid (22n-6) all negatively correlated with the ice-water swimming habit duration (indirect measure of adaptive acclimatization to cold). This suggests that uptake of these fatty acids from circulation and their utilization could be energetically more effective and therefore preferentially used in the cold-acclimatized individuals during the acute cold exposure. Interestingly, LA was in fact the most highly (>6-fold) induced fatty acid in BAT of mice exposed to cold (4 °C) for 48 h [7]. In addition, several other LA-derived metabolites that correlated with ice-water swimming habit duration (DGLA, adrenic acid and arachidonic acid) were also significantly increased in the cold-stimulated BAT [7]. In humans, baseline circulating levels of LA seem to be significantly lower in BAT-positive individuals than in BAT-negative controls, but its levels increase in response to cold exposure only in the BAT-positive group [9]. In contrast, baseline LA levels correlated positively with ice-water swimming activity duration in our cohort (data not shown), and while its levels increased after cold exposure in 10 out of 14 participants of our study, we found that the individual changes were in negative correlation with the duration of ice-water swimming habit. Others have shown that supplementation of conjugated LA to obese mice increases the expression of browning and inflammatory markers in white fat [23]. Furthermore, supplementation of mice with ALA-bio-fortified butter to induce obesity prevented the obesity-induced decline in thermogenic capacity (body temperature) during 3 h acute exposure to 4 °C, reduced the whitening and pro-inflammatory changes in BAT, and increased expression of mitochondrial biogenesis markers in BAT as compared to mice supplemented with conventional butter or margarine [24]. LA and ALA-derived fatty acids also seem to affect BAT thermogenic potential. Treatment of primary brown preadipocytes with EPA increased the expression of brown fat markers as well as basal, uncoupled and maximal oxygen consumption rate [25]. Similar results were obtained in obese mice and HIB 1B cells treated with EPA [26]. On the other hand, arachidonic acid suppresses the brown phenotype in differentiating hMADS cells [27]. Interestingly, LA is a precursor in the synthesis of the lipokine 12,13-diHOME, which has been shown to increase in circulation of both humans and mice in response to 1-h cold exposure and to activate BAT by stimulating the fatty acid uptake [28].

Notably, ice-water swimming-induced change in all the aforementioned fatty acids enriched in the LA and ALA pathway correlated positively with the cold-induced change in parathyroid hormone, which is one of the emerging potential mediators of the thermogenic process [10,29,30]. PTH has direct lipolytic action and we have previously shown it is markedly induced by ice-water swimming in humans [10]. Interestingly, long-chain polyunsaturated fatty acids, especially eicosapentaenoic and docosahexaenoic acids, seem to directly activate PTH type 1 receptor in bone, stimulating extracellular signal-regulated kinase (ERK) and Protein kinase B phosphorylation [31].

By comparing the list of metabolites changed by acute cold stress and those significantly correlating with cold-acclimatization level (ice-water swimming duration/frequency) or anthropometric/circulating parameters of metabolic health, we attempted to identify several metabolites that could have some role in the regulation of metabolic homeostasis during the thermogenic process in response to cold exposure. Interestingly, this group included several amino acids or their derivates, further supporting their potential role in the adaptive thermogenic process in humans. For example, cold-induced change in methionine was positively associated with ice-water swimming habit duration as well as with metabolic health of the ice-water swimmers (negative correlation with insulin resistance index—HOMA-IR). There are reports indicating that dietary methionine restriction has beneficial effects on metabolic health, including insulin sensitivity, and that already 6 days of methionine restriction induces significant increase in energy expenditure, which was at least in part dependent on fibroblast growth factor 21 [32]. The effects on energy expenditure become larger over time, with 30% increase observed after 8 weeks of methionine restriction. This effect seems to be dependent on uncoupling protein 1, and the outcome of the restriction is a profound reduction of adiposity [32,33].

Interestingly, cold-induced change in lysophosphatidylcholine (LPC) 18:2, which contains one chain of linoleic acid, was associated negatively with both ice-water swimming habit duration and metabolic health (positive correlation with atherogenic index). Importantly, lower systemic levels of LPC 18:2 were identified as a novel predictor for impaired glucose tolerance and type 2 diabetes [34]. Furthermore, LPC 18:2 was one of the most markedly increased lipids in subcutaneous and visceral adipose tissue during the browning process induced by 10 days of β3-adrenergic agonist stimulation in mice [35]. 

Finally, we were also able to identify one metabolite, N-lactoyl-Tryptophan (belonging to n-acyl-alpha amino acids), that: (i) was regulated by acute cold, (ii) correlated with ice-water swimming frequency or duration, and (iii) correlated with the cold-induced changes in thyroid hormones (free T4) and PTH. These hormones might have a role in the thermogenic process as discussed in our previous work [10]. It is interesting that there is very little evidence about the function or role of the N-lactoyl-amino acids, but one study identified the process of their synthesis from lactate and amino acids by protease cytosolic nonspecific dipeptidase 2 by reverse proteolysis and hypothesized that this group of metabolites may represent a large portion of the unidentified metabolites that are regulated by exercise or other stimuli [36]. Recently, several N-lactoyl-amino acids have been linked to mitochondrial function [37], which is a crucial aspect of the thermogenic process efficiency.

We also need to acknowledge the limitations of this work. Annotation of metabolites in our untargeted metabolomic analysis was only putative and thus other, targeted methods would be needed to validate the metabolite identity. Furthermore, smaller sample size and imbalanced gender ratio, which arises from the unique study population and protocol, may have limited the statistical power of the analysis. Our results therefore need to be confirmed by further studies. However, we believe that these are important preliminary findings that that might bring focus to potentially important candidate metabolic pathways and thus promote and advance research in the molecular physiology of the thermogenic processes in humans.

## 4. Materials and Methods

### 4.1. Study Population and Protocol

We studied 14 middle-aged ice-water swimmers (gender: 12M/2F, age: 48.9 ± 9.3 y, BMI: 29.7 ± 4.3 kg/m^2^), who were acclimatized to regular outdoor swimming in cold water during winter and were able to endure at least 10 min of swimming in <5 °C cold water. The study protocol and population are described in more detail in our previous publication [10]. We collected blood samples from a cubital vein indoors (room temperature) approximately 1 h before and 10–30 min after swimming for 10–15 min in an ice-cold river (2.6 °C water). The cold exposure began with approximately 30 min period at outdoor temperature (0 °C) in light clothing (swimsuit, T-shirt), followed by swimming in the Danube River in Bratislava, Slovakia (February), wearing a swimsuit (no thermal protection) and a head cover. One participant without plasma sample after ice-water swimming (unsuccessful collection) was excluded from this work. We aimed to avoid any bias related to sample collection and processing. All samples were collected and processed on the same day and within closest time frame possible between participants. We found no correlation between sample collection order and ice-water swimming-induced changes in circulating glucose, insulin, thyroxine, TSH or PTH levels (*p* > 0.05). Food intake prior to the event was not restricted, but participants were asked to refrain from food consumption before the blood collection after swimming. In the following month (average daily air temperature of sampling period: 7.2 °C), volunteers visited our clinical unit after an overnight fast for additional blood sampling and assessment of body composition (bioelectrical impedance, Omron BF511, Omron, Kyoto, Japan), blood pressure and pulse (Omron 907, Omron, Kyoto, Japan) and cold-hardening habits (questionnaire). One participant was unable to attend the phenotyping, therefore the sample and data are missing. Circulating parathyroid hormone (PTH), thyroid stimulating hormone (TSH), free and total thyroxine (T4), total/HDL cholesterol, triglycerides, glucose, insulin, high-sensitivity C-reactive protein (hsCRP), alanine transaminase (ALT) and aspartate transaminase (AST) levels were measured in a certified biochemical laboratory (Unilabs Slovakia s.r.o., Bratislava, Slovakia) by standardized methods using ADVIACentaur Immunoassay and ADVIA Chemistry systems (Siemens, Germany). Atherogenic index of plasma was calculated using formula log ((plasma triglycerides)/plasma HDL cholesterol).

### 4.2. Metabolome Analysis

Polar metabolites of plasma were extracted by mixing 20 μL of plasma with 180 μL of 80% methanol. Upon 1 h incubation at 4 °C, clear extracts were obtained by centrifugation. Non-targeted metabolomics analysis of extracts was performed by flow-injection—time-of-flight mass spectrometry on an Agilent 6550 QTOF system [38]. The instrument was set to scan in full MS at 1.4 Hz in negative ionization, 4 GHz High Res mode, from 50 to 1000 m/z. The solvent was 60:40 isopropanol:water supplemented with 1 mM NH4F at pH 9.0, as well as 10 nM hexakis(1H, 1H, 3H-tetrafluoropropoxy)phosphazine and 80 nM taurochloric acid for online mass calibration. The injection sequence was randomized. Data was acquired in profile mode, centroided and analyzed with Matlab (The Mathworks, Natick, MA, USA). Missing values were filled by recursion in the raw data. Upon identification of consensus centroids across all samples, ions were putatively annotated by accurate mass and isotopic patterns. Starting from the HMDB v4.0 database [39], we generated a list of expected ions including deprotonated, fluorinated, and all major adducts found under these conditions. All formulas matching the measured mass within a mass tolerance of 0.001 Da were enumerated. As this method does not employ chromatographic separation or in-depth MS2 characterization, it is not possible to distinguish between compounds with identical molecular formula. The confidence of annotation reflects Level 4, but in practice in the case of intermediates of primary metabolism it is higher because they are the most abundant metabolites in cells. This resulted in a matrix with 973 putatively annotated ions [11]. Ion intensities were normalized by quantile normalization to compensate for slight variations in the sample amount.

### 4.3. Statistical Analysis

A priori power analysis using circulating markers of thermogenesis measured in this population prior to metabolomic analysis [10] was performed with the G*power v.3.1 [40]. This analysis revealed that to detect significant change in serum inorganic phosphate and parathyroid hormone, study needs to include 5 and 13 individuals, respectively. The probability of type I error (α) was set to 0.05, power (1-β) to 0.80 and expected correlation to 0.4. Cold-induced changes in circulating metabolites were analyzed by Wilcoxon matched pairs signed-rank test after evaluation of normal distribution showing that more than 60% of metabolites did not have normal data distribution. False discovery rate (FDR)-adjusted p-value was calculated by Benjamini–Hochberg correction. Pathway analysis was performed in RaMP [15] and Metaboanalyst 5.0 [12]. Enrichment over-representation analysis of metabolites from correlation analyses was performed in Metaboanalyst 5.0 [12] using the SMPDB Pathway-associated metabolites sets library, and enrichment analysis of annotations for HMDB taxonomy groups was performed in MBROLE 2.0 [41]. Multivariate correlation analysis was performed in JMP SAS 4.0.2 (SAS Institute Inc., Cary, NC, USA) using Spearman’s correlation and significance was set to Spearman’s correlation coefficient Rho > 0.50 or Rho < −0.50. Individual correlations were confirmed by Pearson’s linear regression as specified in the results. Principal component analysis (PCA) and hierarchical clustering analysis were performed in Orange 3.20.0 [42]. Venn diagram was created using InteractiVenn [43].

## 5. Conclusions

In conclusion, our metabolomic data analysis of plasma from acutely cold-exposed cold-acclimatized individuals in relationship to the individual cold exposure frequency and duration as well as metabolic health markers offers an important insight into the systemic adaptive thermogenic processes in humans potentially activated/reflected by the regulated metabolites. Specifically, our data support the role of metabolic pathways related to amino acids, linoleic and α-linolenic acids, which seem to be affected not only by acute cold but also regular cold exposure as well as metabolic phenotype. Therefore, they might play a role in the metabolic adaptations to cold exposure that might provide metabolic benefits to the cold-acclimatized individuals.

## Data Availability

The data presented in this study are openly available in Mendeley Data at doi:10.17632/8fyjd9yrpf.1, Kovanicova, Zuzana; Karhanek, Miloslav; Kurdiova, Timea; Balaz, Miroslav; Wolfrum, Christian; Ukropcova, Barbara; Ukropec, Jozef (2021), “Plasma metabolome cold exposure”, Mendeley Data, V1. Access link https://data.mendeley.com/datasets/8fyjd9yrpf/draft?a=8f865066-31e0-4dc1-a1d8-db27a7cb94e2 (accessed on 10 August 2021).

## Supplementary Materials

The following are available online at https://www.mdpi.com/article/10.3390/metabo11090619/s1, Figure S1: Distance map of the clustering of metabolites significantly changed after ice-water swimming (Wilcoxon matched pairs signed-rank test with Benjamini–Hochberg correction for multiple comparison) in plasma from ice-water swimmers, Table S1: List of significantly regulated plasma metabolites after ice-water swimming (IWS), Table S2: Correlations between ice-water swimming (IWS)-induced changes in plasma metabolites and the IWS duration or frequency (acclimatization level), Table S3: Correlations between ice-water swimming (IWS)-induced changes in metabolites from linoleic and alpha-linolenic acid metabolism pathway and IWS duration (acclimatization level) and IWS-induced change in serum parathyroid hormone (PTH), Table S4: Correlations between ice-water swimming (IWS)-induced changes in plasma metabolites and body mass index (BMI). Data were analyzed using Spearman’s correlation, data with −0.5 > R > 0.5 are shown, Table S5: Correlations between ice-water swimming (IWS)-induced changes in plasma metabolites and Homeostatic Model Assessment for Insulin Resistance index (HOMA-IR), Table S6: Correlations between ice-water swimming (IWS)-induced changes in plasma metabolites and atherogenic index of plasma, Table S7: Correlations between cold-changed plasma metabolites and anthropometric and circulating parameters of metabolic phenotype.
