# Supplementary material for: Metabolomic Analysis Reveals Changes in Plasma Metabolites in Response to Acute Cold Stress and Their Relationships to Metabolic Health in Cold-Acclimatized Humans
Source: Metabolites. 2021 Sep 12;11(9):619. doi: 10.3390/metabo11090619 (PMC8468536; doi:10.3390/metabo11090619)
Supplement: Supplementary file 1 [file metabolites-11-00619-s001.zip › Figure S1.pdf]

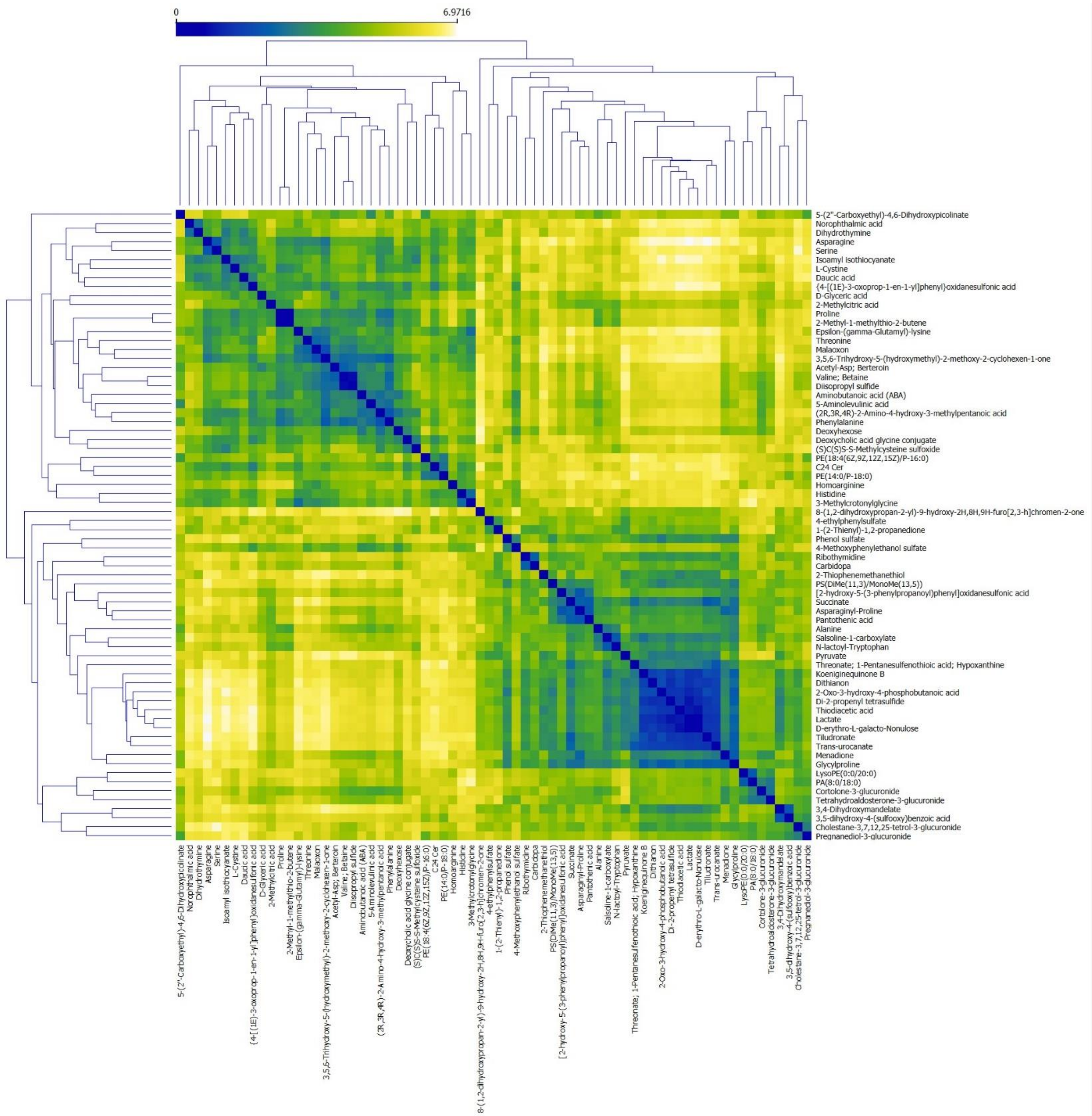

Figure S1. Distance map of the clustering of metabolites significantly changed after ice-water swimming (Wilcoxon matched pairs signed-rank test with Benjamini–Hochberg correction for multiple comparison) in plasma from ice-water swimmers.
